# Mapping the Early Dispersal Patterns of SARS-CoV-2 Omicron BA.4 and BA.5 Subvariants in the Absence of Travel Restrictions and Testing at the Borders in Europe

**DOI:** 10.3390/v15010133

**Published:** 2022-12-31

**Authors:** Evangelia Georgia Kostaki, Elias Mossialos, Ioulia Tseti, Petros P. Sfikakis, Dimitrios Paraskevis

**Affiliations:** 1 Department of Hygiene, Epidemiology and Medical Statistics, Medical School, National and Kapodistrian University of Athens, 11527 Athens, Greece; 2 Department of Health Policy, London School of Economics and Political Science, London WC2A 2AE, UK; 3 Institute of Global Health Innovation, Imperial College London, London SW7 2AZ, UK; 4 Uni-Pharma S.A., 14564 Kifissia, Greece; 5 1st Department of Propaedeutic Internal Medicine, Medical School, National and Kapodistrian University of Athens, 11527 Athens, Greece

**Keywords:** SARS-CoV-2, molecular epidemiology, BA.4, BA.5, omicron subvariants, dispersal patterns

## Abstract

The circulation of SARS-CoV-2 omicron BA.4 and BA.5 subvariants with enhanced transmissibility and capacity for immune evasion resulted in a recent pandemic wave that began in April–May of 2022. We performed a statistical phylogeographic study that aimed to define the cross-border transmission patterns of BA.4 and BA.5 at the earliest stages of virus dispersal. Our sample included all BA.4 and BA.5 sequences that were publicly available in the GISAID database through mid-May 2022. Viral dispersal patterns were inferred using maximum likelihood phylogenetic trees with bootstrap support. We identified South Africa as the major source of both BA.4 and BA.5 that migrated to other continents. By contrast, we detected no significant export of these subvariants from Europe. Belgium was identified as a major hub for BA.4 transmission within Europe, while Portugal and Israel were identified as major sources of BA.5. Western and Northern European countries exhibited the highest rates of cross-border transmission, as did several popular tourist destinations in Southern and Central/Western Europe. Our study provides a detailed map of the early dispersal patterns of two highly transmissible SARS-CoV-2 omicron subvariants at a time when there was an overall relaxation of public health measures in Europe.

## 1. Introduction

SARS-CoV-2 was first identified in Wuhan, China in late 2019 [1,2,3]. Since that time, this emerging virus has developed into a worldwide pandemic, and most experts believe that SARS-CoV-2 will continue to circulate in the human population for many years into the future. New SARS-CoV-2 variants with altered genetic makeup and different biological properties were detected within a few months after the emergence of the parent strain. Several variants of concern (VOCs) have proliferated due to natural selection and have gradually replaced virus strains that were previously in circulation, including SARS-CoV-2 alpha, beta, gamma, delta, and omicron variants. The omicron variant and subvariants exhibit increased transmissibility and are capable of evading both naturally acquired and vaccine-induced immune responses that protected against earlier virus variants [4,5,6,7].

While the emergence of the SARS-CoV-2 omicron variant significantly increased the incidence of COVID-19, these new infections were less likely to result in severe illness. Genetic divergence is one of the major hallmarks of the omicron variant, which now includes several distinct subvariants [8,9,10]. Particularly, the original omicron variant (BA.1) that was described first in South Africa differed by more than 30 mutations on the spike protein with respect to the original strain. After the initial emergence of BA.1, several subvariants such as BA.2, BA.4, and BA.5 detected in South Africa, including additional mutations that further increased their immune evasion capacity, were responsible for a subsequent virus surge in Europe and globally. BA.2 included eight mutations in the spike protein compared to BA.1, and relative to BA.2, BA.4 and BA.5 have the spike mutations 69–70 deletion, L452R, F486V, and the wild-type amino acid Q493.

Apart from the high genetic divergence of the omicron variants compared to all other previously circulating VOCs, the subvariants show extensive divergence within the omicron clade [11,12]. The initial virus dispersal featured subvariants BA.1 and BA.1.1; this was followed by several major subvariant families, including BA.2, BA.4, and BA.5 [13]. Omicron subvariants were associated with pandemic waves that began in November 2021 and continued through the summer months of 2022 [14]. The latest pandemic wave was dominated by BA.4 and BA.5, which are variants with the highest transmissibility and capacity for immune escape [13,15,16,17,18,19]. Omicron subvariants were first detected in South Africa and spread quickly throughout the world, effectively replacing the delta variant and all previously circulating VOCs [20]. The global pattern and dynamics of virus dispersal differ greatly depending on geographic origin [14]. To date, no detailed information has become available that focuses specifically on the transmission patterns of the different omicron subvariants.

This study aimed to investigate both the intra-European and international dispersal patterns of the SARS-CoV-2 BA.4 and BA.5 subvariants via statistical phylogeographic analysis. Specifically, our goal was to elucidate pathways of viral transmission at the earliest time points during April–May 2022, an interval that coincides with the relaxation of public health measures and travel bans designed to limit the transmission of SARS-CoV-2. An investigation of viral transmission patterns within this specific time interval may permit us to identify dispersal hotspots and thus provide insight into how to direct efforts designed to prevent future pandemic spread.

## 2. Materials and Methods

### 2.1. SARS-CoV-2 Sequences

Our study included all SARS-CoV-2 BA.4 and BA.5 sequences sampled from the time of their emergence until 10 May 2022, that were available in the GISAID database (Available online: https://gisaid.org/; accessed on 16 May 2022) [14]. Specifically, we downloaded 1515 BA.4 (Tables S1 and S3) and 866 BA.5 sequences (Tables S2 and S4) that had been completed by this date. We confirmed that all sequences collected from the GISAID database were of the SARS-CoV-2 lineage using the pangolin webtool (Available online: https://cov-lineages.org/pangolin.html; accessed on 17 May 2022). The level of agreement between pangolin webtool and lineage information available at GISAID was 100% for both subvariants. Multiple sequence alignments were performed using the multithreaded version of MAFFT (XSEDE) available from the cyberinfrastructure for phylogenetic research (The CIPRES Science Gateway, v3.3; Available online: https://www.phylo.org/; accessed on 8 September 2022). After the exclusion of seven BA.4 and seven BA.5 sequences that were of low quality, we included 1508 BA.4 and 859 BA.5 sequences with lengths of 29,368 and 29,377 nucleotides (nt), respectively. For this study, fully anonymized nucleotide sequences were retrospectively analyzed; thus, no written informed consent was required.

### 2.2. Phylogenetic Analysis

Phylogenetic analysis is a molecular epidemiology method used to identify patterns of epidemic spread and is based on the analyses of DNA/protein sequences. Therefore, we performed a phylogenetic analysis of SARS-CoV-2 BA.4 and BA.5 sequences to explore their global dispersal patterns. Sequences of each subvariant were examined separately and evaluated by phylogenetic analysis with bootstrap support using the maximum likelihood method (RAxML v8.1.15) with the GTR + G nucleotide substitution model [21]. Phylogenetic reliability was estimated by bootstrap analysis (RAxML v8.1.15). All evaluations were performed at The CIPRES Science Gateway (v3.3). We used FigTree (v1.4) (Available online: http://tree.bio.ed.ac.uk/software/figtree/; accessed on 8 September 2022) for the visualization and annotation of the phylogenetic trees.

### 2.3. Phylogeographic Analysis

Phylogeographic analysis is another molecular epidemiology method used to track viral transmission pathways and monitor virus migration by means of viral phylogenies. This method has been applied to several epidemics. We conducted two phylogeographic analyses in which BA.4 and BA.5 sequences were divided into groups based on their detection in individual countries or continents. This permitted us to estimate the number of viral migration events occurring between different countries and between continents. For the analysis performed at the country level, sequences from the United Kingdom (UK) were grouped into four geographic regions (England, Northern Ireland, Scotland, and Wales). Because sequences from South Africa were overrepresented in both BA.4 and BA.5 subvariant samples, we performed downsampling and created and analyzed three different datasets for each subvariant that contained 100, 150, or 300 (295 for BA.5) sequences from this country, including those with the earliest sampling dates. Specifically, downsampling was not performed after a random selection of sequences from South Africa, but those with the earliest sampling dates were retained in each dataset. This was done so that the number of sequences from South Africa would not overwhelm the contributions from the next largest source of sequence data. We note that this condition was not fulfilled in the dataset that included 100 sequences from South Africa. Countries that provided fewer than 10 sequences were omitted from these datasets. Migration events between the different countries and continents were inferred from bootstrap-reconstructed phylogenetic trees (*n* = 450 for datasets with 295 or 300 South African sequences, and *n* = 400 for datasets with 150 or 100 South African sequences) via character reconstruction using maximum parsimony methods (PAUP*4.0 program) [22]. By this method, a character representing the country or continent of sampling was assigned at the tips of the bootstrap trees, and the algorithm reconstructed the ancestral states in the tree’s nodes by using the criterion of parsimony (minimum number of state changes). We then used a statistical phylogeography approach to identify potentially significant transmission pathways for each dataset. Statistical phylogeography permitted us to determine whether the inferred migration events were or were not likely to occur by chance, with complete geographic mixing (panmixia) used to represent the null hypothesis as described in detail in previous publications [23,24,25]. In brief, the panmixis hypothesis assumes that an infected individual has the same probability to transmit the virus to any other non-infected individual. To simulate a phylogenetic tree inferred from a population fulfilling this hypothesis, we performed a random reshuffling of the taxa at the tips of the bootstrap trees in Mesquite program [26]. A transmission pathway was considered significant if the distributions of the migration events inferred from the bootstrap trees before and after the reshuffling of the taxa at their tips differed significantly (Mann–Whitney U test, Bonferroni-adjusted *p*-values). More details about these methods have been provided elsewhere [23,24,25]. Our final report was limited to the significant transmission pathways inferred among the European countries and Israel that were the same in all BA.4 datasets as well as in the datasets that included 300 and 150 South African BA.5 sequences. The resulting pathways were categorized into two groups according to their *p*-values. Pathways with *p*-values ≤ 5.00 × 10^−50^ in all BA.4 datasets and datasets with 300 and 150 South African BA.5 sequences were identified as the most significant ones.

## 3. Results

Our study included 1508 and 859 high-quality BA.4 and BA.5 sequences, respectively, that were downloaded from the GISAID database [14]. While most of these sequences were from virus samples collected in South Africa (BA.4: *n* = 928, 61.5%; BA.5: *n* = 295, 34.3%), a comparatively large number of BA.5 sequences were also collected from Germany (*n* = 143, 16.6%) and Portugal (*n* = 133, 15.5%). The distribution of BA.4 and BA.5 sequences by country and continent is shown in Table 1 and Table 2. Because we aimed to identify BA.4 and BA.5 transmission patterns at the earliest stage of the recent pandemic wave, we included all available sequences that were collected before May 2022 (Tables S1 and S2) [14,27].

### 3.1. Viral Transmission Patterns of BA.4 Subvariant

As described in the Materials and Methods section, phylogeographic analysis was initiated using several subsets of sequences from South Africa, which was the country with the largest number of available sequences for both subvariants. To generate these subsets, we performed downsampling and created three distinct datasets that included 100, 150, and ~300 of the earliest sampled South African sequences. In addition, only countries from which more than nine sequences were collected were included in the final analysis. Phylogeographic analysis of BA.4 revealed consistent findings across all three datasets. Although our initial findings suggested that several migration events may have originated in Europe, the statistical phylogeographic analysis revealed that the extent of outgoing viral mobility (i.e., export) from Europe was largely insignificant. Evaluation of all three datasets revealed that South Africa was the major source of BA.4 dispersal to both Australia and Israel. Analysis of the downsampled datasets with 100 and 150 South African sequences revealed that South Africa was also the main source of the virus that was dispersed within Europe. The United States (US) was also a source of virus dispersal to all destinations including Israel as determined by our evaluation of the smaller datasets. Although our findings suggested significant export of virus from Australia and Israel, fewer than one migration event was detected for each of their respective destinations.

**Figure 1:**
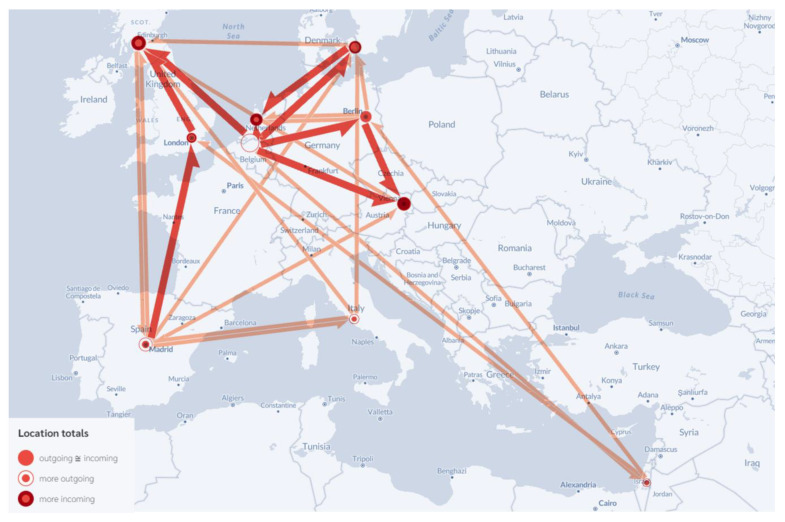
Significant early dispersal pathways of the SARS-CoV-2 BA.4 subvariant across Europe. The red lines indicate the most significant viral migration events (*p*-values ≤ 5.00 × 10^−50^); the direction of each arrow documents the destination. Dots represent capital cities and not the place of residence of infected individuals. The color of each dot indicates the relationship between the points at the beginning and end of each arrow, including nearly equal ingoing and outgoing, more outgoing, or more incoming for each geographic locality. FlowmapBlue tool was used to visualize the data as a flow map (Available online: https://flowmap.blue/; accessed on 6 October 2022).

Phylogeographic analysis of BA.4 dispersal is shown in Figure 1. Our analysis across all three datasets revealed that Belgium, Spain, Germany, and Italy were major sources of BA.4 virus dispersal, as they exported the virus to six, five, three, and three countries, respectively. Denmark, Scotland, and Israel each exported BA.4 to two countries, while England and the Netherlands were each identified as the source of BA.4 virus dispersal to one country. We detected no significant virus export from Austria. The major destinations for virus dispersal were Scotland, Denmark, and Austria, which imported BA.4 from five, four, and four countries, respectively. This was followed by Germany, the Netherlands, England, and Spain, each importing BA.4 from three, three, two, and two countries, respectively. While both Israel and Italy imported BA.4 from one country each, no BA.4 virus was imported into Belgium. Statistical phylogeography revealed that the most significant events included transmission pathways that originated in Belgium, specifically those from Belgium to the Netherlands, Germany, Denmark, Austria, and Scotland. Other significant events included transmission from England to Scotland, from Germany to Austria, from Denmark to the Netherlands, and from Spain to England. The findings reported here are shown in Figure 1.

### 3.2. Viral Transmission Patterns of BA.5 Subvariant

A similar analysis was performed for the BA.5 variant that dominated the recent pandemic wave in Europe and worldwide. Our findings are based on an analysis of two of the three BA.5 datasets that included 300 and 150 sequences from South Africa. Our results revealed that South Africa was the major source of BA.5 virus dispersal to both Israel and the US (as determined using the dataset with 300 South African sequences); this finding was extended to Europe when evaluating the dataset in which South African sequences were downsampled to 150. While the US was a major source of the BA.5 virus transferred to Europe and South Africa, no significant virus export from Europe to other regions was detected using both datasets. By contrast, BA.5 virus from Israel was transported to both Europe and the US (as per the evaluation of two datasets).

**Figure 2:**
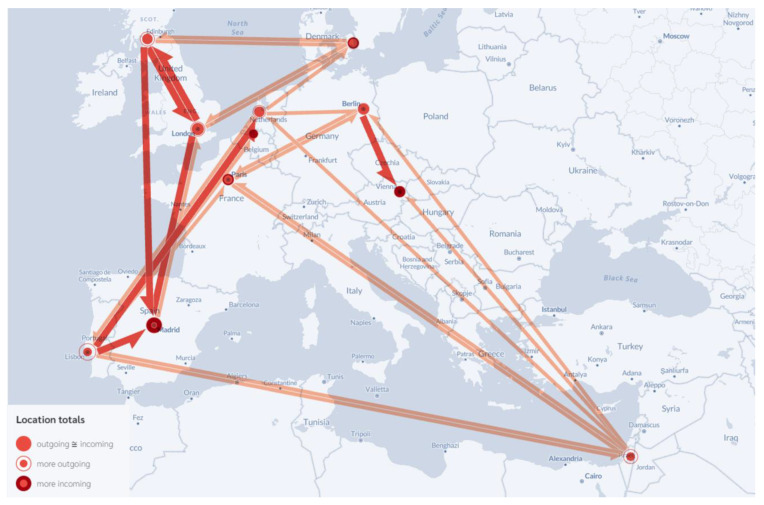
Significant early dispersal pathways of SARS-CoV-2 BA.5 subvariant across Europe. The color and classification of countries are as described in Figure 1. Flow map was created by using FlowmapBlue tool.

The phylogeographic analysis of BA.5 is shown in Figure 2. Our findings revealed that Israel and Portugal each exported BA.5 virus to five other countries and thus represent major sources of BA.5 virus dispersal. The Netherlands, England, and Scotland exported BA.5 virus to four, three, and three countries, respectively, while Denmark, France, and Germany exported BA.5 virus to two countries each. While BA.5 export from Spain targeted one country, no significant export from Austria or Belgium was detected. The most significant transmission pathways were those from Portugal to Spain and the Netherlands, from England to Scotland and Spain, from Germany to Austria, and from Scotland to England and Spain. With respect to imported BA.5 infections, no major differences were observed among the countries under evaluation because most of them received the infectious virus from two or three countries only.

## 4. Discussion

Newly emerging SARS-CoV-2 omicron subvariants BA.4 and BA.5 were responsible for the latest summer pandemic wave in Europe and throughout the world [13]. Both subvariants emerged in South Africa [13,20,27] and quickly migrated elsewhere because of their enhanced transmissibility compared to the previously circulating subvariants BA.1 and BA.2 [19,27]. To enhance the sensitivity, our study sample was limited to viruses that were collected in the GISAID database that were dated through 10 May 2022. This set included viral sequences that were in circulation at the early stages of the recent pandemic wave. Previous studies have shown that local infections that were attributed to one of the new, highly transmissible SARS-CoV-2 variants could be readily transferred across borders during the early stages of the BA.4 and BA.5 pandemic waves.

Our phylogeographic analysis focused on the earliest available sampled sequences and revealed that both BA.4 and BA.5 subvariants originated in South Africa. While the US was also identified as a significant source of viral transmission, we observed no significant virus export from Europe. These results suggest that, compared to other nations of similar size and economic development, the population of Europe had limited mobility and was less likely to travel to other areas during the earliest stages of this pandemic wave.

Patterns of BA.4 and BA.5 transmission varied substantially within Europe. Transmission of BA.4 was comparatively low. Belgium was the epicenter of BA.4 virus dispersal in Europe, followed by Spain. Additional pathways were mapped mostly in Western Europe and the UK. Notably, BA.4 was most frequently transmitted between neighboring countries in Western Europe and the UK except for pathways both to and from Spain, Italy, and Israel. Virus transmission to and from Spain and Italy might be directly related to the influx of tourists, given that both countries are popular destinations for individuals from Western and Northern Europe.

BA.5 dispersal followed a completely different pattern. Portugal and Israel were among the major sources of BA.5 dispersal. Both countries exported the virus to several different areas; Portugal was the source of BA.5 for two of the major dispersal pathways within Europe. We note that BA.5 was detected earlier in Portugal and spread more effectively from this country than from anywhere else in the European continent [14]. BA.5 was detected in Portugal at the beginning of April and became the dominant variant 1 month thereafter, at the beginning of May; this subvariant was the source of a rather large pandemic wave in this country (World Health Organization; Available online: https://www.WHO.int; accessed on 15 September 2022). Therefore, the pattern of BA.5 export from Portugal resulted from its earlier introduction and dispersal within a country that had recently lifted travel restrictions. In Israel, BA.5 was first detected in early May, which was somewhat later than in Portugal. BA.5 dominated thereafter [14] with no major differences observed when compared with most of the other European countries. Additional pathways of BA.5 dispersal included the UK, the Netherlands, Germany, and Denmark.

Interestingly, and in contrast to its role in exporting BA.4, Belgium had no significant role in promoting the dispersal of BA.5. Similarly, Portugal played no major role in the dispersal of BA.4. Some pathways supported the dispersal of both subvariants, although BA.5 emerged later and at higher levels than BA.4. These pathways included connections between England and Scotland, Germany and Austria, and Spain and England. Overall, the major travel hubs in Western and Northern Europe and Spain were associated with the major pathways of both BA.4 and BA.5 dispersal. Similar results were reported for the initial dissemination of SARS-CoV-2 during the first pandemic wave from February to March 2020. At that time, COVID-19-associated deaths in Western and Northern Europe were reported earlier and at higher rates than in Central and Eastern Europe (World Health Organization; Available online: https://www.WHO.int; accessed on 15 September 2022). Although colder weather and indoor ventilation can contribute to the transmissibility of SARS-CoV-2 [28], the most recent virus surges took place during the summer months across Europe [14]; these findings suggest that these factors have no significant impact on the dissemination of new virus variants.

Our study has some limitations. A potential limitation is the inability to produce real-time data about the dispersal patterns of SARS-CoV-2; however, this is not feasible for molecular epidemiology studies. Our findings were based on the phylogeographic analyses of SARS-CoV-2 sequences that were available in the GISAID database at the early stages of BA.4 and BA.5 dispersal. One cannot rule out that sampling may occur more frequently and thus be biased towards countries with more intensive genomic surveillance programs. However, this will probably have little to no effect on the inferred transmission pathways within Europe, as there are no substantial differences between countries with respect to the intensity of genomic surveillance. To increase the sensitivity of our analysis, we included all of the available full-length viral sequences from this database and created datasets with different numbers of sequences from the countries providing most of the available data. The possibility that some countries might have been left out of our study due to late submission of their sequences to GISAID cannot be excluded; however, our results were in accordance with the epidemiological findings about the dispersal patterns of BA.4 and BA.5 in Europe.

Taken together, the results of our phylogeographic analysis permitted us to map the early dispersal patterns of the two recently emerged omicron subvariants both globally and within Europe. Omicron subvariants are genetically diverse, a feature that facilitates their evasion of antiviral immunity that was acquired through vaccination or natural infection with another variant [9,10,11,12,15,17,18,19]. By the time both BA.4 and BA.5 emerged, a large fraction of the population in Europe and other Western countries had been vaccinated or had recovered from an earlier SARS-CoV-2 infection. Therefore, infection with an omicron variant was generally associated with a lower risk of developing the more severe forms of COVID-19 compared to those that developed in response to viruses of previous clades. These observations led to a relaxation of public health measures, a more tolerant attitude toward individual behaviors, and the elimination of strict travel restrictions within Europe. Specifically, restrictions on non-essential travel were lifted, and COVID-19 testing was no longer required for crossing national borders within the European Union (EU). Moreover, many EU countries eliminated the temporary restriction on non-essential travel into the EU by individuals who had been vaccinated, provided that the traveler had received the final dose of the primary series at least 14 days but no more than 9 months before their arrival or had received an additional booster dose. The latter decision was set forth by the European Council on 22 February 2022 and applied thereafter throughout Europe. Thus, the early stages of BA.4 and BA.5 dispersal in Europe took place under conditions and travel restrictions that were similar to those in place during the pre-COVID era. This might be in contrast to events that took place during the first waves of the pandemic when individual mobility and travel restrictions were in force [29] and summer tourism remained limited [30].

The interval beginning in February 2022 and continuing through the summer season represents a new situation with respect to public health measures and individual behavior during a holiday period. We aimed to investigate the rates and pathways of BA.4 and BA.5 transmission during summer in Europe when travel restrictions had returned to their pre-pandemic state. Among our results, we found that the major source or the “founder” country differs according to the nature and dynamics of each subvariant. For example, Portugal was identified as a major source of BA.5 because of its earlier dominance compared to other European nations. In addition to these “founder” or source countries, we found that major travel hubs in Western Europe were also associated with high levels of virus dispersal. By contrast, virus mobility seems to be delayed in Eastern and Southern European countries compared to what was observed in their Western and Northern European counterparts. We anticipate that these patterns will remain consistent during subsequent pandemic waves and may provide an early indication of the transmission patterns of new SARS-CoV-2 variants as well as other pathogenic respiratory viruses.

## Figures and Tables

**Table 1:** Total number of SARS-CoV-2 BA.4 sequences from GISAID database sampled until 10 May 2022, categorized by continent, country, and region of sampling. Sequences of low quality were excluded.

Continent of Sampling	Country of Sampling	Region of Sampling	Sequences, *n* (%)
Europe	Austria		80 (5.2)
	Belgium		38 (2.5)
	Denmark		47 (3.1)
	France		8 (0.5)
	Germany		35 (2.3)
	Ireland		2 (0.1)
	Italy		24 (1.6)
	Luxembourg		1 (0.1)
	Netherlands		15 (1.0)
	Spain		10 (0.7)
	Sweden		1 (0.1)
	Switzerland		2 (0.1)
	United Kingdom	England	94 (6.2)
		Northern Ireland	1 (0.1)
		Scotland	31 (2.1)
		Wales	2 (0.1)
Sub-total			391 (25.8)
Oceania	Australia		19 (1.3)
	New Zealand		1 (0.1)
Sub-total			20 (1.4)
Asia	Israel		32 (2.1)
	Singapore		3 (0.2)
Sub-total			35 (2.3)
Africa	Botswana		1 (0.1)
	South Africa		928 (61.5)
Sub-total			929 (61.6)
America	Canada		7 (0.5)
	Chile		1 (0.1)
	United States		125 (8.3)
Sub-total			133 (8.9)
Total	22		1508 (100.0)

**Table 2:** Total number of SARS-CoV-2 BA.5 sequences from GISAID database sampled until 10 May 2022, categorized by continent, country, and region of sampling. Sequences of low quality were excluded.

Continent of Sampling	Country of Sampling	Region of Sampling	Sequences, *n* (%)
Europe	Austria		24 (2.8)
	Belgium		12 (1.4)
	Denmark		17 (2.0)
	France		20 (2.3)
	Germany		143 (16.6)
	Iceland		4 (0.5)
	Italy		5 (0.6)
	Netherlands		10 (1.2)
	Norway		1 (0.1)
	Portugal		133 (15.5)
	Spain		20 (2.3)
	Switzerland		2 (0.2)
	United Kingdom	England	54 (6.3)
		Northern Ireland	2 (0.2)
		Scotland	25 (2.9)
Sub-total			472 (54.9)
Oceania	Australia		6 (0.8)
Asia	Israel		20 (2.3)
	China		2 (0.2)
Sub-total			22 (2.5)
Africa	South Africa		295 (34.3)
America	Canada		1 (0.1)
	United States		63 (7.4)
Sub-total			64 (7.5)
Total	19		859 (100.0)

## Data Availability

Data used in the context of this study were publicly available in the GISAID database [Available online: https://gisaid.org/ (accessed on 16 May 2022)]. Accession numbers for nucleotide sequences are provided in Tables S3 and S4.

## Supplementary Materials

The following supporting information can be downloaded at: https://www.mdpi.com/article/10.3390/v15010133/s1, Table S1: Earliest and latest sampling dates for the SARS-CoV-2 BA.4 sequences under study; Table S2: Earliest and latest sampling dates for the SARS-CoV-2 BA.5 sequences under study; Table S3: Accession numbers for the SARS-CoV-2 BA.4 sequences under study; Table S4: Accession numbers for the SARS-CoV-2 BA.5 sequences under study.
