# Supplementary material for: Mapping the Early Dispersal Patterns of SARS-CoV-2 Omicron BA.4 and BA.5 Subvariants in the Absence of Travel Restrictions and Testing at the Borders in Europe
Source: Viruses. 2022 Dec 31;15(1):133. doi: 10.3390/v15010133 (PMC9865345; doi:10.3390/v15010133)
Supplement: Supplementary file 1 [file viruses-15-00133-s001.zip › viruses-2070387-supplementary.pdf]

Supplementary materials for:

Mapping the early dispersal patterns of SARS-CoV-2 omicron BA.4 and BA.5 subvariants in the absence of travel restrictions and testing at the borders in Europe

Kostaki EG, Mossialos E, Tseti I, Sfikakis PP, Paraskevis D

Contents:

Supplementary Table S1. Earliest and latest sampling dates for the SARS-CoV-2 BA.4 sequences under study……………………………………………………………………………………………..……….**2**

Supplementary Table S2. Earliest and latest sampling dates for the SARS-CoV-2 BA.5 sequences under study……………………………………………………………………………………………...….**3**

Supplementary Table S3. Accession numbers for the SARS-CoV-2 BA.4 sequences under study ……………………………………………………………………………………………...……….**4**

Supplementary Table S4. Accession numbers for the SARS-CoV-2 BA.5 sequences under study …………………………………………………………………………………………………….**12**

Supplementary Table S1. Earliest and latest sampling dates for the SARS-Cov-2 BA.4 sequences under study.

Country of sampling	Region of sampling	Earliest sampling date	Latest sampling date
Austria		05/04/2022	05/05/2022
Belgium		29/03/2022	10/05/2022
Denmark		02/03/2022	08/05/2022
France		30/03/2022	02/05/2022
Germany		12/04/2022	09/05/2022
Ireland		30/04/2022	01/05/2022
Italy		20/04/2022	05/05/2022
Luxembourg		25/04/2022	25/04/2022
Netherlands		10/04/2022	05/05/2022
Spain		07/04/2022	06/05/2022
Sweden		04/05/2022	04/05/2022
Switzerland		04/04/2022	28/04/2022
United Kingdom	England	22/03/2022	10/05/2022
	Northern Ireland	21/04/2022	21/04/2022
	Scotland	22/03/2022	06/05/2022
	Wales	11/04/2022	03/05/2022
Australia		31/03/2022	04/05/2022
New Zealand		27/04/2022	27/04/2022
Israel		20/04/2022	08/05/2022
Singapore		11/04/2022	06/05/2022
Botswana		12/04/2022	12/04/2022
South Africa		10/01/2022	09/05/2022
Canada		04/04/2022	30/04/2022
Chile		19/04/2022	19/04/2022
USA		30/03/2022	09/05/2022

Supplementary Table S2. Earliest and latest sampling dates for the SARS-Cov-2 BA.5 sequences under study.

Country of sampling	Region of sampling	Earliest sampling date	Latest sampling date
Austria		20/04/2022	05/05/2022
Belgium		24/04/2022	10/05/2022
Denmark		15/04/2022	08/05/2022
France		28/03/2022	02/05/2022
Germany		16/03/2022	06/05/2022
Iceland		28/04/2022	30/04/2022
Italy		03/05/2022	03/05/2022
Netherlands		25/04/2022	06/05/2022
Norway		12/04/2022	12/04/2022
Portugal		30/03/2022	26/04/2022
Spain		15/04/2022	05/05/2022
Switzerland		11/04/2022	28/04/2022
United Kingdom	England	14/04/2022	09/05/2022
	Northern Ireland	21/04/2022	22/04/2022
	Scotland	14/04/2022	06/05/2022
Australia		13/04/2022	04/05/2022
Israel		21/04/2022	08/05/2022
China		08/04/2022	23/04/2022
South Africa		25/02/2022	09/05/2022
Canada		15/04/2022	15/04/2022
USA		29/03/2022	10/05/2022

Supplementary Table S3. Accession numbers for the SARS-CoV-2 BA.4 sequences under study.

10807016	11867431	12006291	12043279
10860989	11867434	12006301	12043280
11270126	11867443	12006305	12043281
11275475	11867448	12006308	12043283
11331250	11867453	12006310	12043286
11542270	11867456	12006312	12043288
11542287	11867458	12012872	12043289
11542474	11867461	12012877	12043291
11542550	11867470	12012886	12043292
11542567	11867475	12012887	12043293
11542603	11867486	12012891	12043296
11550739	11867495	12012896	12067585
11604519	11867499	12012910	12086839
11645323	11867510	12012915	12097314
11674410	11867524	12012922	12097316
11674411	11867525	12012935	12097317
11674413	11867526	12012939	12097318
11674425	11867527	12012941	12097319
11674426	11873073	12012944	12097322
11674430	11903031	12013570	12097323
11674431	11903035	12043216	12097326
11674432	11903059	12043218	12097328
11674443	11913288	12043222	12097329
11674447	11984860	12043225	12097330
11674448	11984862	12043226	12097332
11674784	11984863	12043228	12097333
11752668	11988685	12043229	12097334
11763475	11990462	12043230	12097337
11763478	11994300	12043231	12097338
11763480	11999052	12043234	12097340
11763481	11999053	12043235	12097343
11763491	11999054	12043236	12097344
11763502	11999055	12043238	12097346
11773266	11999056	12043241	12097348
11773269	11999057	12043243	12097349
11773271	11999058	12043244	12097352
11773278	11999065	12043260	12097353
11825926	11999078	12043261	12097354
11825931	12006222	12043263	12097357
11825937	12006223	12043264	12097379
11825942	12006230	12043265	12097391
11832686	12006240	12043266	12097393
11866393	12006243	12043267	12099407
11867344	12006245	12043268	12109331
11867346	12006251	12043269	12116582
11867366	12006259	12043270	12119232
11867372	12006273	12043271	12122771
11867409	12006286	12043272	12123784
11867426	12006288	12043273	12130073
11867428	12006289	12043278	12139399

12149337	12274007	12293148	12307616
12152675	12274009	12293149	12307617
12169006	12274011	12293150	12307618
12169353	12274024	12293154	12307620
12179199	12274030	12293158	12307624
12180585	12274031	12293159	12307626
12180587	12274036	12293160	12307627
12180593	12274037	12293161	12307628
12206542	12274038	12293162	12307630
12208047	12274039	12293267	12307631
12208064	12274041	12293270	12307633
12211242	12274049	12293273	12307634
12212563	12274051	12293281	12307635
12212566	12274056	12293282	12307640
12212568	12274060	12293283	12307641
12212569	12274073	12293284	12307642
12212571	12274074	12293285	12307643
12217097	12274076	12293286	12307645
12221587	12274078	12293287	12307648
12223371	12274083	12293288	12307651
12226685	12274085	12293291	12307653
12226709	12274086	12293292	12307658
12241466	12274088	12293293	12307660
12248508	12274094	12295715	12307663
12252797	12274095	12307541	12307664
12252953	12274097	12307542	12307665
12256369	12274099	12307543	12307670
12264646	12274637	12307544	12307672
12264896	12278927	12307548	12307673
12266791	12278955	12307550	12307674
12267751	12278965	12307551	12307675
12267752	12278967	12307552	12307680
12267754	12278970	12307560	12307683
12267759	12278973	12307561	12307686
12268494	12278989	12307562	12307687
12268495	12280677	12307564	12307690
12268504	12280890	12307568	12307694
12268506	12281895	12307569	12307696
12273981	12281998	12307571	12307698
12273982	12283356	12307575	12307699
12273984	12286266	12307577	12307700
12273986	12292990	12307579	12307704
12273988	12292992	12307580	12307705
12273990	12293081	12307582	12307706
12273992	12293138	12307584	12307709
12273994	12293139	12307604	12307712
12273995	12293140	12307605	12307714
12273997	12293142	12307606	12307715
12274003	12293143	12307607	12307716
12274006	12293144	12307608	12307718

12307719	12401108	12426980	12472142
12307721	12401110	12435606	12472152
12307722	12401111	12436012	12472153
12307723	12401112	12436133	12472154
12317495	12401113	12442764	12472155
12318109	12401114	12445747	12472206
12335014	12401115	12450643	12472350
12339502	12401116	12450648	12473024
12340151	12401117	12450908	12473600
12344221	12401118	12451079	12473605
12347452	12401119	12454576	12473608
12361027	12401127	12456544	12473612
12361049	12401129	12456614	12473861
12368176	12401132	12456676	12473864
12376427	12401134	12456726	12473865
12396843	12401135	12456788	12473867
12401033	12401136	12456790	12473870
12401040	12401137	12456969	12473871
12401041	12401138	12462038	12473872
12401043	12401139	12462123	12473873
12401045	12401140	12465863	12473877
12401047	12401144	12466376	12473878
12401051	12401146	12469584	12473881
12401052	12401148	12469594	12473882
12401055	12401149	12469612	12473883
12401056	12401155	12469632	12473884
12401058	12401156	12469666	12473885
12401059	12401159	12469728	12473889
12401061	12401160	12469747	12474407
12401062	12401165	12469892	12474409
12401063	12401174	12470002	12474410
12401065	12401175	12471213	12474413
12401067	12401176	12471284	12474415
12401068	12401348	12472109	12474417
12401069	12401707	12472111	12474418
12401071	12401714	12472114	12474419
12401072	12401721	12472118	12474423
12401073	12416220	12472123	12474426
12401074	12416794	12472124	12474427
12401079	12421048	12472127	12474428
12401082	12426939	12472128	12474429
12401083	12426942	12472129	12474431
12401085	12426944	12472130	12474433
12401087	12426947	12472131	12474435
12401089	12426952	12472133	12474437
12401091	12426956	12472134	12474438
12401092	12426957	12472136	12474440
12401095	12426958	12472137	12474441
12401096	12426967	12472138	12474443
12401101	12426977	12472139	12474444

12474446	12477027	12515674	12527983
12474448	12477029	12515844	12529661
12474449	12477031	12515952	12531068
12474450	12477033	12516253	12531636
12474451	12477034	12518072	12532190
12474453	12477036	12519987	12533200
12474458	12477037	12519988	12533570
12474459	12477040	12519989	12533695
12474461	12477041	12519993	12533737
12474462	12477725	12519995	12534961
12474463	12478413	12519996	12534967
12474464	12478907	12519999	12534968
12474465	12479048	12520001	12534969
12474468	12481214	12520003	12534971
12474469	12482009	12520005	12534975
12474470	12483994	12520006	12534976
12474472	12484020	12520007	12534977
12474475	12490941	12520009	12534978
12474476	12493665	12520010	12534979
12474478	12496098	12520011	12534981
12474480	12496100	12520015	12534983
12474481	12496101	12520018	12534984
12474483	12496103	12520022	12534985
12474485	12496106	12520023	12534986
12474486	12496108	12520024	12534987
12474487	12496111	12520026	12534989
12474488	12496114	12520027	12534990
12474489	12496121	12520028	12534991
12474490	12496123	12520029	12534992
12475182	12496124	12520030	12534993
12475185	12497122	12520034	12534995
12475853	12497804	12520036	12534996
12476623	12502027	12520037	12534997
12476624	12502253	12520038	12534999
12476993	12502627	12520040	12535000
12476997	12505183	12520042	12535001
12476998	12505602	12520044	12535002
12476999	12505659	12520045	12535031
12477001	12507303	12520046	12535221
12477002	12507726	12520048	12535551
12477003	12507862	12520057	12536335
12477004	12511645	12520058	12536377
12477007	12512307	12520059	12546339
12477008	12512309	12520062	12546776
12477011	12514770	12520064	12547957
12477021	12514937	12520065	12548001
12477022	12515007	12520814	12548012
12477024	12515119	12523958	12548483
12477025	12515126	12523974	12548717
12477026	12515210	12526038	12550282

12553282	12559461	12576104	12587442
12553722	12559462	12576105	12587444
12555769	12559463	12576106	12587445
12555841	12559464	12576108	12587446
12557381	12560039	12576109	12587447
12558222	12560421	12578776	12587448
12558347	12562652	12578777	12587449
12558354	12563502	12580061	12587451
12558608	12563697	12582288	12587452
12558790	12563969	12582817	12587453
12558792	12567767	12585438	12587465
12558793	12570194	12585863	12587879
12558795	12570336	12585869	12587880
12558796	12570506	12585880	12587881
12558797	12571624	12585882	12587882
12558799	12571627	12585883	12587884
12558800	12571630	12585894	12587885
12558801	12573683	12585896	12587886
12558802	12574029	12585897	12587887
12558804	12574074	12585900	12587888
12558806	12574305	12585901	12587889
12558807	12576033	12587328	12587890
12558808	12576034	12587398	12587891
12558810	12576035	12587401	12587892
12558811	12576036	12587403	12587893
12558813	12576037	12587404	12587894
12558815	12576038	12587405	12587895
12558817	12576039	12587406	12587896
12558818	12576040	12587407	12587897
12558821	12576042	12587408	12587898
12558822	12576043	12587409	12587899
12558824	12576044	12587410	12587900
12558825	12576045	12587411	12587901
12558826	12576046	12587415	12587902
12558827	12576047	12587417	12587905
12558829	12576049	12587419	12587906
12558927	12576050	12587420	12589000
12559414	12576051	12587421	12589010
12559417	12576052	12587424	12589020
12559422	12576053	12587427	12589156
12559437	12576054	12587428	12589338
12559438	12576055	12587429	12589890
12559451	12576056	12587431	12590125
12559452	12576093	12587432	12590338
12559453	12576096	12587434	12590371
12559454	12576098	12587435	12590398
12559455	12576099	12587436	12590447
12559456	12576100	12587437	12592810
12559457	12576101	12587438	12593409
12559458	12576103	12587439	12594370

12594933	12607958	12610609	12636857
12595455	12607959	12610610	12637061
12595506	12607965	12610612	12637066
12595673	12607966	12610965	12637322
12596123	12607967	12610991	12637330
12596327	12607968	12610999	12638056
12596867	12607969	12612375	12638091
12597267	12607971	12612666	12638562
12600647	12607972	12613687	12638698
12601588	12607975	12613688	12638712
12604145	12607977	12613766	12638809
12605007	12607978	12618322	12638937
12605011	12607980	12619000	12638991
12605042	12607981	12620864	12639043
12605043	12607982	12621814	12639175
12605102	12607983	12621878	12640456
12605463	12607986	12622491	12640486
12605606	12607987	12622501	12640913
12605687	12607988	12623503	12642583
12605801	12607994	12624687	12642823
12606310	12607995	12624817	12643124
12606334	12607996	12626263	12643898
12606511	12608000	12626309	12644444
12606534	12609354	12627205	12644809
12606668	12609474	12628235	12644815
12606795	12609645	12628241	12644817
12606828	12610054	12628250	12644821
12606834	12610106	12628267	12644835
12606838	12610165	12628303	12644839
12606861	12610277	12629118	12644843
12606881	12610334	12629144	12644849
12606913	12610551	12629153	12644885
12607382	12610560	12629210	12644886
12607392	12610563	12629453	12647216
12607398	12610564	12629568	12647217
12607412	12610569	12629946	12647650
12607935	12610571	12630402	12647695
12607937	12610574	12630820	12647704
12607938	12610580	12630919	12648066
12607939	12610581	12630959	12648815
12607940	12610582	12632184	12648960
12607941	12610583	12632471	12648962
12607944	12610585	12632539	12649098
12607946	12610588	12633086	12649169
12607947	12610589	12633207	12651470
12607949	12610593	12636562	12651471
12607951	12610594	12636596	12651484
12607953	12610597	12636743	12651486
12607954	12610598	12636752	12651488
12607955	12610600	12636843	12651490

12651491	12652826	12673528	12695072
12651492	12652828	12673868	12695285
12651493	12652832	12673961	12696584
12651495	12652837	12674190	12698346
12651496	12652838	12674581	12698363
12651497	12652840	12675976	12698379
12651498	12652843	12677290	12698427
12651499	12652845	12677579	12698430
12651500	12652846	12679072	12698463
12651501	12652847	12679128	12698475
12651585	12652848	12679153	12698480
12651663	12652849	12679171	12698497
12651869	12653326	12679182	12698534
12651975	12653559	12679229	12698555
12652205	12653996	12680051	12698564
12652697	12654786	12680383	12698570
12652708	12654787	12682132	12698633
12652744	12654788	12682277	12698635
12652745	12655488	12682345	12698702
12652746	12657215	12682471	12698710
12652747	12657353	12683313	12699259
12652748	12659666	12683708	12699566
12652752	12659677	12685173	12699568
12652756	12659759	12687955	12699727
12652757	12660066	12687970	12699746
12652770	12660069	12688305	12699750
12652773	12660354	12688770	12699755
12652775	12660374	12688820	12699930
12652776	12660422	12688839	12700044
12652777	12660425	12689375	12700725
12652780	12660430	12689597	12700869
12652782	12660439	12689860	12701327
12652783	12660480	12689861	12701562
12652785	12660487	12690245	12701860
12652786	12660498	12692295	12701930
12652787	12660507	12693857	12701964
12652788	12665547	12694012	12702013
12652790	12665652	12694023	12702023
12652791	12667094	12694101	12702035
12652793	12670312	12694146	12702045
12652796	12670828	12694220	12703161
12652797	12671000	12694256	12704014
12652801	12671280	12694285	12704161
12652802	12671294	12694978	12704609
12652811	12671688	12694992	12705226
12652813	12672007	12695005	12705469
12652815	12672113	12695027	12705546
12652818	12672180	12695028	12705595
12652822	12672213	12695031	12705839
12652825	12673525	12695055	12705840

12705842	12705920	12705999	
12705843	12705921	12706002	
12705844	12705922	12706004	
12705845	12705923	12706005	
12705847	12705934	12706007	
12705848	12705936	12706008	
12705852	12705937	12706344	
12705853	12705938	12706359	
12705854	12705939	12706389	
12705855	12705940	12706401	
12705856	12705941	12706680	
12705857	12705942	12706846	
12705858	12705943	12708421	
12705859	12705944	12708740	
12705860	12705945	12709399	
12705861	12705946		
12705862	12705947		
12705863	12705949		
12705865	12705951		
12705867	12705952		
12705868	12705953		
12705869	12705954		
12705870	12705955		
12705871	12705956		
12705872	12705958		
12705873	12705959		
12705877	12705961		
12705879	12705964		
12705880	12705965		
12705884	12705966		
12705886	12705967		
12705888	12705968		
12705889	12705969		
12705890	12705970		
12705891	12705971		
12705892	12705972		
12705894	12705973		
12705895	12705976		
12705896	12705978		
12705897	12705979		
12705899	12705981		
12705906	12705983		
12705909	12705985		
12705910	12705990		
12705911	12705991		
12705912	12705992		
12705916	12705993		
12705917	12705995		
12705918	12705996		
12705919	12705997		

Supplementary Table S4. Accession numbers for the SARS-CoV-2 BA.5 sequences under study.

11017528	12097321	12252681	12289904
11542465	12097324	12267757	12289911
11542604	12097361	12268493	12289935
11674786	12097362	12268496	12290004
11763507	12097378	12268499	12290110
11763512	12097383	12268503	12291987
11763513	12097392	12268509	12293136
11763514	12097395	12268513	12300640
11763515	12097397	12268514	12307546
11763516	12097401	12273993	12307549
11763528	12097405	12273999	12307553
11763529	12097406	12274002	12307557
11763530	12097407	12274018	12307576
11763533	12097408	12274046	12307609
11763534	12097409	12274070	12307612
11763535	12097410	12278969	12307619
11763536	12112497	12278971	12307629
11817426	12113187	12278976	12307647
11818312	12114808	12278977	12307652
11867415	12127266	12278982	12307654
11867511	12127267	12278983	12307662
11903041	12127324	12278987	12307666
11903042	12127327	12278990	12307671
11903043	12127412	12278992	12307676
11903044	12127418	12278993	12307684
11903045	12127420	12278995	12307685
11903046	12127435	12281109	12307693
11903047	12127456	12281146	12307695
11903048	12127463	12282421	12307707
11903049	12151839	12282880	12307710
11903051	12181265	12283695	12307717
11903052	12181267	12288598	12311863
11903062	12187584	12288613	12315631
11903064	12187920	12289676	12317782
11992074	12188629	12289678	12333300
11999060	12188650	12289715	12334086
12011087	12188674	12289723	12334817
12012871	12188680	12289728	12334819
12012890	12190233	12289733	12339573
12012905	12190609	12289737	12346538
12012919	12194677	12289738	12348018
12014443	12195043	12289741	12348900
12029894	12207803	12289748	12350567
12043282	12210559	12289761	12350568
12043290	12211144	12289865	12350571
12063903	12211851	12289870	12354933
12063929	12212221	12289873	12355213
12085863	12216342	12289899	12356895
12085895	12224559	12289901	12356943
12097320	12237160	12289903	12357300

12358680	12440237	12477009	12520746
12358695	12442758	12477012	12520888
12359690	12456712	12477016	12520982
12359716	12457428	12477017	12523344
12360010	12464658	12477018	12523570
12360686	12464660	12477020	12523699
12361163	12464668	12477039	12525869
12361382	12464676	12477378	12525871
12361559	12464678	12477386	12525877
12395556	12464680	12477408	12525879
12396824	12464685	12477450	12525880
12401036	12464700	12477516	12525881
12401038	12464769	12477557	12525887
12401042	12464772	12477592	12525888
12401044	12464774	12477595	12525891
12401046	12464776	12478418	12525895
12401048	12464782	12489816	12525909
12401049	12464824	12490240	12526117
12401077	12464847	12490468	12526672
12401080	12464893	12490598	12526675
12401081	12464905	12490829	12526758
12401107	12464920	12492164	12526974
12401123	12464927	12495201	12527102
12401126	12464928	12502938	12527337
12401131	12464930	12503210	12528735
12401133	12465068	12513180	12528955
12401142	12465077	12513244	12529089
12401145	12465086	12514451	12534973
12401150	12465112	12514552	12534974
12401153	12465862	12514771	12534980
12401154	12469529	12515490	12535004
12401158	12469675	12515717	12540781
12401161	12469711	12516495	12543177
12401163	12472550	12517340	12546671
12401168	12473106	12517523	12546886
12401169	12473599	12517547	12546954
12401171	12473602	12517692	12547382
12406393	12473610	12517818	12548500
12426941	12473869	12518111	12549316
12426978	12473876	12518135	12557301
12431299	12474422	12518145	12557593
12431300	12474442	12519251	12557952
12431301	12474447	12519392	12558341
12431302	12474460	12519821	12558791
12431303	12474466	12519998	12558798
12431317	12474467	12520002	12558803
12434881	12474471	12520013	12558812
12434896	12474473	12520047	12558816
12434927	12474479	12520053	12558820
12439885	12476995	12520056	12558828

12559411	12570864	12591440	12598764
12559412	12570868	12595717	12598765
12559413	12570894	12595730	12598766
12559416	12570895	12597022	12598768
12559418	12570901	12598476	12598772
12559419	12571623	12598500	12598774
12559420	12571625	12598502	12598775
12559421	12571626	12598527	12598780
12559423	12571628	12598539	12598781
12559424	12571629	12598542	12598782
12559425	12574125	12598547	12598787
12559427	12576041	12598548	12598788
12559428	12576073	12598551	12598791
12559429	12576074	12598553	12598792
12559430	12576075	12598554	12598798
12559431	12576077	12598555	12598799
12559432	12576080	12598557	12598803
12559433	12576083	12598558	12598815
12559434	12576084	12598560	12598825
12559435	12576097	12598561	12598834
12559436	12582143	12598567	12598869
12559439	12585604	12598587	12598872
12559440	12585864	12598594	12598908
12559441	12585887	12598618	12598935
12559442	12585892	12598637	12598936
12559444	12587418	12598643	12598945
12559445	12587422	12598646	12598949
12559446	12587426	12598656	12598963
12559448	12587440	12598675	12598965
12561198	12587441	12598704	12598980
12561199	12587450	12598705	12605106
12561210	12587876	12598715	12606114
12561211	12587877	12598723	12606165
12561212	12587883	12598726	12606170
12561213	12587903	12598730	12606358
12562014	12587904	12598736	12606368
12563489	12588374	12598738	12606501
12563943	12588803	12598739	12606698
12565762	12589272	12598741	12606965
12565792	12589340	12598743	12607074
12565942	12589393	12598748	12607952
12567679	12589394	12598749	12607956
12567712	12589398	12598750	12607974
12568859	12589400	12598752	12607984
12568880	12589402	12598753	12607997
12568930	12589404	12598756	12607998
12570652	12589405	12598757	12609307
12570776	12589406	12598758	12610548
12570845	12589408	12598761	12610549
12570849	12590612	12598762	12610552

12610557	12637237	12671167	12688263
12610561	12637250	12672524	12688266
12610572	12638367	12672529	12688267
12610573	12638474	12672566	12688835
12610587	12639017	12672591	12689598
12610592	12640425	12673390	12690265
12610595	12641024	12673414	12690576
12610602	12642590	12673606	12694033
12610605	12642820	12674056	12694237
12610608	12642821	12674280	12694282
12611442	12642824	12674286	12694985
12611590	12642829	12674332	12694988
12615486	12643339	12674577	12694991
12620608	12644727	12674661	12694993
12620609	12644818	12674669	12694994
12620611	12647435	12674677	12694995
12620614	12647486	12674681	12694996
12620642	12647508	12675136	12694997
12620786	12648721	12675185	12694998
12620815	12649069	12675204	12694999
12620848	12650406	12675291	12695001
12621327	12651494	12675523	12695002
12621507	12652754	12675627	12695003
12623188	12652758	12675653	12695004
12628220	12652759	12675796	12695006
12628227	12652769	12675883	12695007
12629136	12652799	12675969	12695008
12629161	12652817	12676008	12695009
12629178	12652841	12676116	12695010
12629451	12653062	12676357	12695011
12629491	12653641	12676625	12695012
12629930	12654529	12677099	12695013
12630040	12655180	12677333	12695016
12630291	12655363	12677347	12695017
12630456	12655572	12677371	12695018
12630492	12655967	12677397	12695019
12630539	12657253	12677438	12695020
12630738	12657356	12677458	12695021
12632483	12658243	12677468	12695022
12632509	12658266	12677693	12695023
12632568	12659704	12677748	12695026
12633005	12660362	12677777	12695029
12633212	12660377	12678043	12695071
12635851	12665900	12678057	12695073
12635951	12666681	12678310	12696267
12635953	12666729	12678459	12698523
12636044	12666860	12678893	12698536
12636091	12667361	12678905	12698760
12636432	12670050	12685224	12698762
12636801	12671029	12686451	12698780

12699044	12705957		
12699047	12705960		
12699052	12705962		
12699057	12705963		
12699059	12705975		
12699138	12705988		
12699139	12705994		
12699263	12705998		
12699426	12706001		
12699516	12706006		
12699555	12706810		
12699613	12707544		
12699651	12707642		
12699895	12707749		
12699943	12708520		
12699989	12708585		
12700109			
12701260			
12701336			
12701484			
12701993			
12703369			
12703370			
12703371			
12703372			
12703373			
12703375			
12704095			
12704229			
12704254			
12704285			
12704369			
12705536			
12705838			
12705841			
12705850			
12705851			
12705875			
12705876			
12705881			
12705882			
12705883			
12705885			
12705898			
12705901			
12705903			
12705904			
12705907			
12705913			
12705935			
